# Concerning Operation for Abscess of the Brain Associated with Otorrhœa

**Published:** 1907-12-14

**Authors:** 


### Otology.

### CONCERNING OPERATION FOR ABSCESS OF THE BRAIN ASSOCIATED WITH OTORRHŒA.

In more than half the cases of brain abscess the infection originates in the cavities of the middle ear, and in these cases the abscess is nearly always single. Its situation in the brain is very typical indeed, yet it is seldom possible to determine the position of an abscess clinically by the so-called localising symptoms. Indeed it is often quite impossible to say whether the suspected abscess is in the temporo-sphenoidal lobe or in the cerebellum. Up to the present time the only practical and reliable method of ascertaining the position of the lesion in the brain is that of following the track of infection from the middle-ear cavities.

The first step is to establish the existence of middle-ear disease. Sometimes the presence of otorrhœa is overlooked. This may be explained by the frequent diminution in the discharge from the meatus with the onset of intra-cranial symptoms, or by the disregard of a condition which may have originated in infancy and hitherto caused no pain. At other times the oversight is due to inaccurate otoscopic observations. A cholesteatomatous mass in the depth of the auditory canal may appear to be cerumen, and may completely obscure valuable evidence of disease in the tympanic cavity. Having established the existence of middle-ear disease we next review the symptoms suggesting the spread of infection.

1. *Septic manifestations.*—The patient becomes cheerless and easily tired. He loses appetite and becomes constipated. Sometimes there is a rigor and pyrexia at the onset, but in a few days the temperature becomes normal or subnormal; but in spite of this the general symptoms of sepsis continue.

2. *Intra-cranial symptoms.*—Headache is very severe; continuous and paroxysmal vomiting may be caused by sudden change in posture. Dizziness is also a common symptom. The patient loses interest in his surroundings, becomes slow in expression of his thoughts, and exhibits, perhaps, some confusion of ideas.

Although the pulse is not always below sixty, it is remarkable how uniformly slow its rate is; indeed, of the above mentioned manifestations, continuous headache and slowness of pulse, associated with otorrhœa, represent the predominant features of the case; and just as in acute abdominal affections, the pulse rate by its increased frequency determines the urgency of the condition, so in intra-cranial disorders the pulse rate, by its tardiness, denotes the serious aspect of the disease.

Again and again it has been shown that the prognosis of brain abscess is much more favourable when the operation has been performed on account of continuous headache and slowness of pulse, than when the operation has been delayed until definite localising symptoms declare themselves. Moreover delay involves a risk in many cases of sudden death, without any localising symptoms arising. The operation is therefore indicated at that stage when the patient, being obviously seriously ill, the diagnosis of brain abscess is merely a probable one.

### 1. Temporo-sphenoidal Abscess.

Of the methods of operating, that of exposing the temporo-sphenoidal lobe laterally, through the squamous portion of the temporal bone, above the auricle, is least often successful, and is now regarded as obsolete. According to present-day methods, the trephine has been replaced by the gouge, hammer and bone forceps, and the operation is performed by opening the abscess through the middle-ear cavities.

After fully opening the mastoid antrum and accessory cells in the manner described in previous issues of The Hospital*, the roof of the cavity is removed, and the dura mater of the middle cranial fossa exposed. Frequently an extra-dural abscess is met with. If the surface of the dura is covered with granulations, or with a purulent exudate, or is soft, and perforated by a fistulous track, the dura should be incised in the diseased area, and the adjacent brain explored with a narrow, sharp-pointed knife. 1. The dura about to be incised should be disinfected. 2. The incision in the dura should be free, lest in the abscence of adhesions at the seat of puncture, between the dura and the brain, pus leak from the punctured abscess into the intra-dural space. 3. The brain should never be explored with a blunt instrument. 4. In puncturing the brain the knife should be passed vertically upwards into the temporo-sphenoidal lobe, and not for greater depth than one inch. Lateral pressure of the blade will allow pus to flow out.

When the abscess has been found the opening should be enlarged, and the cavity freely drained with rubber tube of not less than $\frac{3}{8}$ in. to $\frac{1}{2}$ in. bore. This should be secured by suture, and the wound will need to be dressed daily, the patency of the tube being consistently maintained.

Drainage will be continued with tubes of decreasing diameter for at least a couple of weeks, and the patient kept under constant observation until the mastoid cavity has completely cicatrised. Even after healing is complete, the patient should be enjoined to report himself at regular intervals for a year.

### 2. Cerebellar Abscess.

*Route* 1.—Should exploration of the temporo-sphenoidal lobe, carried out on the above lines, fail to discover pus, the cerebellum must be explored. To do so the posterior cranial should be freely opened, if this has not indeed already been done, by removal of the posterior wall of the antrum and mastoid cavity. The lateral sinus will probably first come into view. Very accurate anatomical knowledge of the variations in this part of the temporal bone is necessary to expose properly the cerebellum by this route. To reach the usual site of a cerebellar abscess the dura mater between the lateral sinus and the posterior semi-circular canal is laid bare, for it is over this area that the dura is generally infected and adherent to

* October 26 and November 9.

the anterior surface of the cerebellar hemisphere. After free incision of the dura, as in the case of temporo-sphenoidal exploration, the cerebellum is punctured with the knife, in a direction backwards and inwards, keeping to the horizontal plane of the head, and in such a manner that the handle of the knife lies at an angle of 45° with the side of the patient's face. The point of the knife should not penetrate, as a rule, deeper than three-quarters of an inch in this direction.

*Route* 2.—In the presence of an abnormally prominent lateral sinus there may be very little space between the sinus and the labyrinth, through which to expose the cerebellum. When this space is so small that efficient drainage through it could not be established, then the abscess must be sought for from the occipital surface below and behind the mastoid process, and below and behind the genu of the lateral sinus. This area having been exposed by removal of bone with cutting forceps, the dura is incised and the cerebellum punctured in a direction upwards, forwards, and inwards, parallel with the posterior surface of the petrous. In other words, the puncture is made behind the genu of the lateral sinus instead of in front of it. There is more uncertainty in finding the abscess by this route, and there is a greater risk of pus leaking into the intradural space, on account of the absence of adhesions in the area penetrated.

### 3. Cerebellar Abscess With Lateral Sinus Thrombosis.

*Route* 3.—When thrombosis of the lateral sinus is found, and symptoms of brain abscess coexist, the cerebellum is explored through the inner wall of the sinus, to which in such cases it is generally adherent. Under these circumstances, the abscess is nearer the outer part of the lateral hemisphere, and the knife is passed directly inwards through the open sinus. In other respects, as regards the brain lesion, the treatment does not differ from that already described under temporo-sphenoidal abscess.

